# Model test study on the mechanical response of the deep buried tunnel lining

**DOI:** 10.1038/s41598-024-63438-5

**Published:** 2024-06-04

**Authors:** Longyan Duan, Jimeng Feng, Jiadai Song, Shiyu Yao

**Affiliations:** 1 Key Laboratory of Transportation Tunnel Engineering, Ministry of Education, Southwest Jiaotong University, Chengdu, 610031 China; 2 School of Civil Engineering, Southwest Jiaotong University, Chengdu, 610031 China

**Keywords:** Weak surrounding rock, Mechanical response, Deep buried tunnel lining, Model test, Civil engineering, Composites

## Abstract

Deep-buried tunnels with weak surrounding rock are frequently encountered issues in traffic engineering. It plays an important role in the excavation process and the project operation. This paper applies the theoretical analysis and laboratory test related to four different conditions in terms of their thickness to determine the mechanical response of deep-buried tunnel lining. Then, the energy dissipative structure theory is employed to explain the experimental results. This paper has made the following achievements: firstly, it is found that the toughness of the secondary lining was found to be often the most important indicator of tunnel safety, with better-toughness linings having higher tensile strength and crack resistance. Secondly, it suggests that the inclusion of steel reinforcement in the concrete lining can effectively improve the secondary lining toughness. Finally, it proves that the more ductile liner had more energy, higher load-carrying capacity, and was better able to maintain the overall stability of the structure.

## Introduction

Fei et al.^1^ pointed out that more than 70% of tunnels in China are experiencing degradation problems, such as water leakage, cracking, spalling and lining block falling. Therefore, many studies have been conducted on issues related to tunnel lining. Some studies have focused on minimizing the construction risk of underground works. However, further research on the stability of the excavation face will have a significant impact on the overall performance of the shallow tunnel. Tunnel excavation stability has become the most concerned issue in tunnel structure research^2^. The stability of the tunnel is directly affected by the compressive deformation of the rock or soil directly in front of the excavation face^3^.

The formation of material damage occurs as a result of changes in its internal microstructure, which manifests itself in the expansion and evolution of microcracks. When microstructural changes accumulate to a certain level, deformation and damage occur at the macro level^4,5^. Excavation-induced damage plays a crucial role in determining the stability of a rock mass. Understanding the characteristics of damage evolution during excavation of underground structures can help in designing reinforcement systems to mitigate the instability of the surrounding rock mass^6,7^.

Tunnel excavation is a typical unloading process that breaks the original stress equilibrium and leads to stress redistribution. During this process, the mechanical, hydraulic and geochemical properties of the surrounding rock will change^8^. After deep underground excavation, the three-dimensional (3D) stress state of the surrounding rock undergoes complex changes, such as stress unloading and concentration, accompanied by deterioration of mechanical properties and changes in the ductile–brittle behavior of the rock mass, which is known as perimeter rock damage^9^. Many researchers have investigated the role of the post-destructive behavior of rock masses on the destructive or plastic regions and deformation around underground excavations^10^. The durability of tunnels also requires close attention to damaged perimeter rock. Under the interaction of internal and external factors, internal corrosion and surface damage (cracks, spalling of blocks, etc.) can occur to the lining structure. All these factors eventually lead to damage or failure of the tunnel structure^11^.

With the continuous development of urban space in China and around the world, tunnel construction is often challenged by the diversity of geotechnical conditions, such as expansive soils, soft soils, karst formations, water-rich formations, and various mixed formations^12^. The surrounding rock may be affected by external factors^13^. For example, under permafrost conditions, the structural integrity of the lining system is affected by cracks, and the tunnel structure is a safety risk due to prolonged exposure to freeze–thaw cycles^14^. Groundwater infiltration weakens the mechanical properties of rocks to varying degrees, increasing shear cracking, and even at high loading rates, water increases the fracture behavior of rocks^15–17^. One type of rock that is extremely sensitive to water is expansive mudstone, and it has been found that the expansion force of the surrounding rock is the main load that affects the lining stresses^18^.

After the tunnel is built, the tunnel structure is affected by the soft rock creep effect. The pressure on the structure can easily reach its upper limit, leading to problems such as cracking of the lining, extrusion or falling blocks, causing safety hazards^19^. In tunneling operations, tunnel structures are susceptible to complex groundwater and geological conditions, construction disturbances around the tunnel, and other factors. Especially in some special cases, it may be challenging for the conventional lining to ensure the safety and stability of tunnels in the long term^20,21^.

The lining is the main structure of a tunnel, used to withstand the pressure of the surrounding rock, and its thickness represents its load-bearing capacity. Defects in the thickness of the tunnel lining will have a direct impact on the long-term stability of the tunnel project^22^. If the design lining is thin, it is prone to cracking during operation. Cracks in the lining will not only reduce the durability and impermeability of the tunnel structure but will also reduce the load-carrying capacity and safety of the structure^23^.

For large section tunnels or tunnels with soft surrounding rocks, thicker secondary lining should be designed to ensure the safety and stability of the tunnel. It is controversial whether the thicker lining is favorable to the durability of the tunnel. Therefore, Feng et al.^24^ conducted a series of modeling tests to explore the correlation between lining stiffness and tunnel durability.

This paper applies the laboratory model test to analyze the different types of the lining of the tunnel under deeply buried condition. The paper consists of the following four parts, the experimental design is established in the first part, the second section discusses the test situation and experimental data, the third section conducts an in-depth discussion of the problem, and the conclusion is presented in the fourth section.

## Experimental design

The design of the model test mainly consists of the simulation method, the similarity ratio design, the material selection and test and the arrangement of monitoring points.

### Simulation method

**Figure 1:**
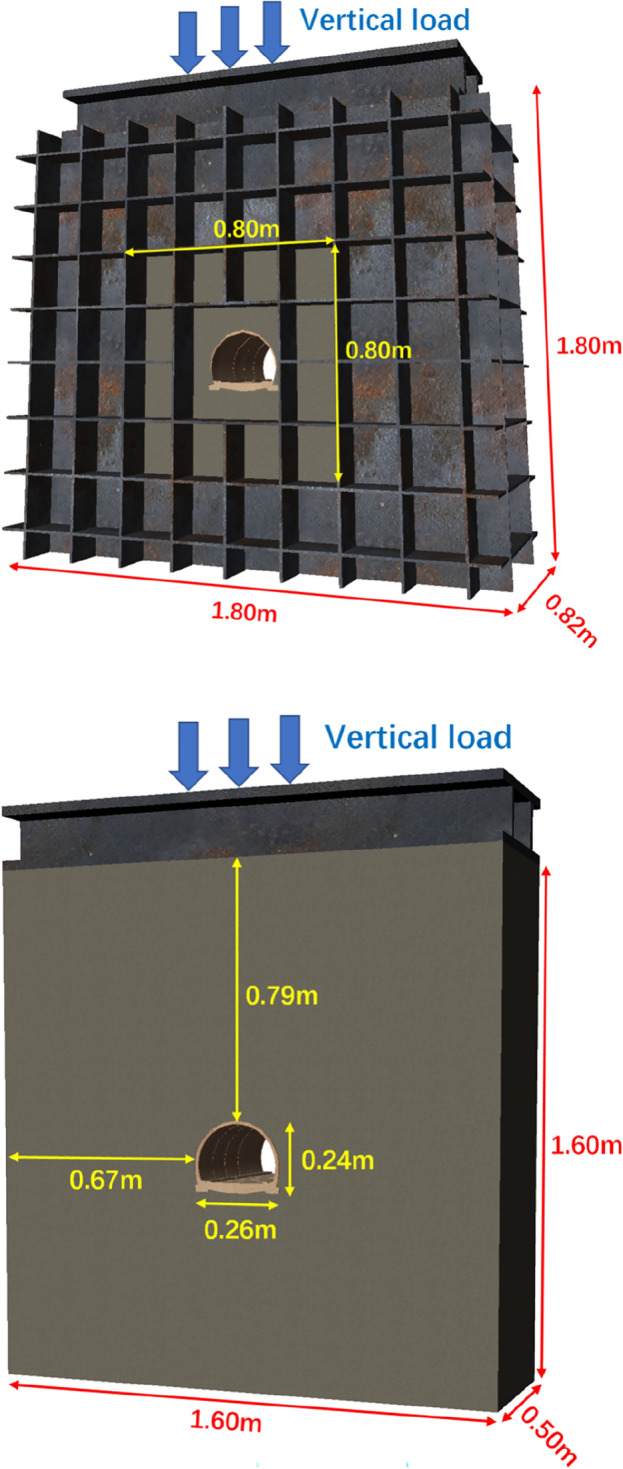
The experimental model box.

The geometric similarity ratio is 1:50. As the test box shown in Fig. 1, top covered plate was designed as loaded beam, it can move up and down and control the load by MTS. In order to meet the plane strain condition, the outside of the plate was designed with rib reinforcement to prevent deformation.

The situation that the horizontal force is often greater than the vertical force in the deep buried tunnel. Different horizontal forces have an impact on the stress of the supporting structure. However, this paper mainly discusses the safety performance of different supporting structures, and simulates the process of excavation and support first and then continuous deterioration. The load is only one aspect of the impact, mainly the load transfer of the deterioration of the surrounding rock itself. Therefore, the influence of the horizontal force value of the model boundary on the simulation results is small. Therefore, in this model test, only the loading of vertical load is considered.

### Similarity ratio design

The physical quantities’ similarity ratios are estimated on the basis of the indoor model test and the basic principle of Buckingham theorem. Table 1 summarizes the related parameter ratios.

**Table 1:** Parameter ratio of similitude.

Name	Proportion	Name	Proportion
Geometric proportion C_l_	50	volume-weight ratio C_γ_	1
Stress ratio C_σ_	50	elastic modulus ratio C_E_	50
Cohesion ratio C_c_	50	internal friction angle ratio C_φ_	1
Strain ratio C_ε_	1	displacement ratio C_u_	50
Boundary force ratio C_X_	50	physical ratio C_g_	1

### Material selection and test

The implementation of indoor modeling tests is based on the theory of similarity, which uses artificial materials with engineering properties similar to those of natural rock. In this experiment, the lining of the tunnel is simulated by gypsum and water, the surrounding rock is simulated by silt and clay. The experimental mechanical parameters are presented in Table 2. As can be seen from Table 2, with the increase of moisture content, the elastic modulus, cohesion and internal friction angle of Clay are obviously reduced. Therefore, it appears to be suitable for simulating the surrounding rock. In this experimental study, the diameter of surrounding rock applied to be twice that of the tunnel. The bolt and steel are simulated by aluminum wire coated with sand.

**Table 2:** The mechanical parameters of the surrounding rock material.

Materials	Moisture content (%)	Elastic modulus (MPa)	Internal friction angle	Cohesion (MPa)	Poisson ratio
Silty sand 1	5.01	11.8	45.1	0.019	0.31
Silty sand 2	11.02	12.1	45.3	0.029	0.32
Clay 1	9.68	20.5	46.93	0.031	0.32
Clay 2	20.49	3.2	34.15	0.065	0.36

The tunnel structure mainly consists of structures such as initial support, anchors, steel arches and secondary lining. The support parameters are as follows:

1. Sprayed concrete was C25 concrete with a thickness of 25 cm; gypsum with a thickness of 4 mm was used for the model test.
2. The steel arch is made of I18 I-beam, laid in the whole ring; the model test is simulated by 3 mm diameter copper bar, which is plastic and easy to bend into the required tunnel section shape.
3. The length of the anchor is 3 m, and the ring direction * longitudinal direction is arranged according to 1.0 m*1.0 m; in the test, the copper wire with a diameter 6 cm is used to simulate the rod, and it is arranged according to 6 cm*6 cm.

For different types of secondary lining, gypsum is used for modeling in the experiment. The water-paste ratio of gypsum determines the physical parameters of the model, and many researches have been done by the previous researchers, and the configuration of the modeled gypsum in this experiment will be referred to the research results of previous papers. The material parameters of gypsum are shown in Table 3. The mechanical parameters of tunnel materials are shown in Table 4.

**Table 3:** Mechanical parameters of plaster.

Number	Gypsum ratio	Moisture content (%)	Elastic modulus (MPa)	compressive strength (MPa)	Poisson ratio
M1	1:1.5	0	398	4.7	0.21
M2	1:1.4	0	323	4.3	0.21
M3	1:1.3	0	297	3.2	0.21
M4	1:1.2	0	269	2.4	0.21

**Table 4:** Mechanical parameters of tunnel.

Tunnel structure	Prototype		Model	
	Parameter	EI (MPa m^4^) EA (MPa m^2^)	Parameter	Corresponding prototype
Primary support	25cmthick C25 Sprayed concrete	26.4	6mmthick M4	30.2
Bolt	3 m, 1 m × 1 m	2.38 × 10^–3^	6 cm, 6 × 6 cm	1.96 × 10^–3^
Steel frame	I18, Whole ring arrangement	3.486	3 mm diameter	3.479
Lining 1	60cmthick C35 concrete	540	1.5cmthick M1	568.8
Lining 2	60cmthick C35 reinforced concrete	540	1.5cmthick M1, Steel wire mesh	568.8
Lining 3	40cmthick C30 reinforced concrete	160	1cmthick M2, Steel wire mesh	168.23

### Arrangement of monitoring points

Four measurement sections are set up for this model test, and the section measurements are contained:

1. Ground displacement during excavation;
2. Lining deformation during excavation;
3. Perimeter rock pressure during excavation;
4. Lining model strain.

**Figure 2:**
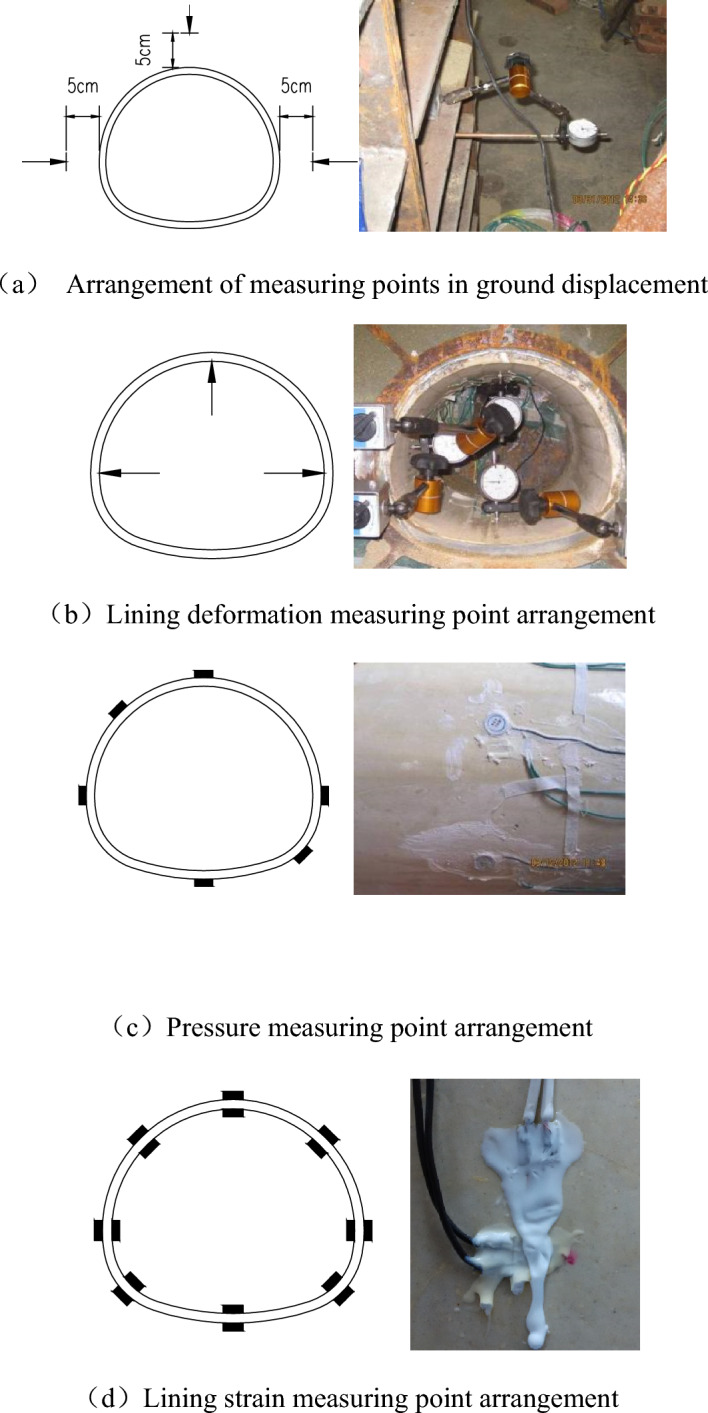
Locations of measurement points.

The arrangements of the monitoring points are shown in Fig. 2. As is presented in Fig. 2, the displacement of the ground is measured by the test tube, which is composed of a copper sleeve and a thin copper rod. The deformation of the lining is measured by strain gauges and the strain gauge soil pressure box is applied to estimate the pressure of surrounding rock. Then, the strain gauge data were collected through the strain collection box.

### Test procedure

Test procedure in this paper includes the following 4 steps:

1. 200 kN vertical force is loaded to simulate of the 500m-formation pressure after filling the dry clay.
2. Observing the displacement of the upper cover plate, open the excavation mouth to start digging when it is stable.
3. After excavating the tunnel contour and completing the initial support construction, the prefabricated secondary lining is embedded and then grouted to maintain the tightness of the initial support and secondary lining. After the above steps were completed, measuring elements such as the surrounding rock earth pressure box were installed, test tubes were placed for measuring ground displacements, and the test system was debugged in time.
4. Water is poured from the top of silt and clay to simulate the deterioration process of surrounding rock after water is poured. After the soil is saturated, the deterioration of surrounding rock is completed.

### Test condition

In order to make clear the different situations of the time of the support and lining stiffness influence on surrounding rock, this paper carries out different simulations respect to four different conditions.

The four different lining types are no second lining structure and three other types of concrete lining with different thicknesses, the initial support consists of shotcrete and steel mesh, and the concrete lining is divided into reinforced concrete and ordinary plain concrete lining. The details of the condition are presented in Table 5.

**Table 5:** The conditions.

Condition no.	Lining design	Remarks
W1	Lining 1	Reinforced concrete lining with thick thickness
W2	Lining 2	Plain concrete lining with thick thickness
W3	Lining 3	Reinforced concrete lining with thin thickness
W4	No secondary lining	Only do the initial support

## Test situation and result analysis

### Tunnel excavation state description

**Figure 3:**
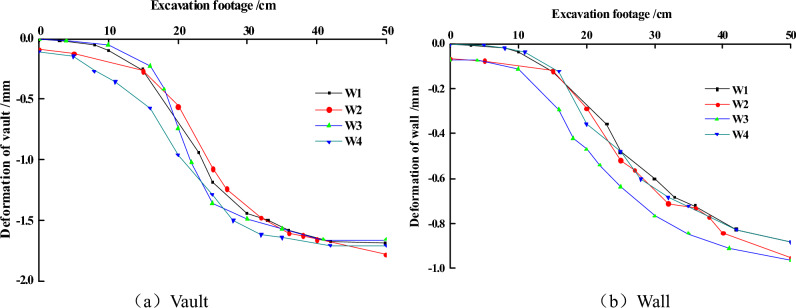
The displacement of rock mass during excavation process.

In the process of tunnel excavation, the ground displacement is the most accurate data to reflect the stability of the tunnel. Figure 3 exhibits the changing of the deformation of the measurement points located in the ground of the arch crown and the side wall during the hole’s excavation are shown. From the figure, it is obvious that when the excavation face did not reach the point of measurement, the displacement of the measuring point already began to appear. With the excavation face near the point, the development of displacement is gradually increasing. When the excavation face passed through the measuring point, the increase of the displacement gradually slowed down. As a whole, the change trajectory of W1–W4 are very similar. This means that the influence of the filling soil can be ignored when filling soil into the model box in the experiment.

### Description of state of deterioration of surrounding rock

The deterioration of surrounding rock was simulated by watering the surrounding rock, the surrounding rock would gradually saturated with water and the strength of the clay would gradually decrease. The secondary lining has a large deformation during the surrounding rock deterioration. The tunnel deformation tends to increase with the development of cracks, then, the secondary lining will not be able to continue to carry the pressure.

**Figure 4:**
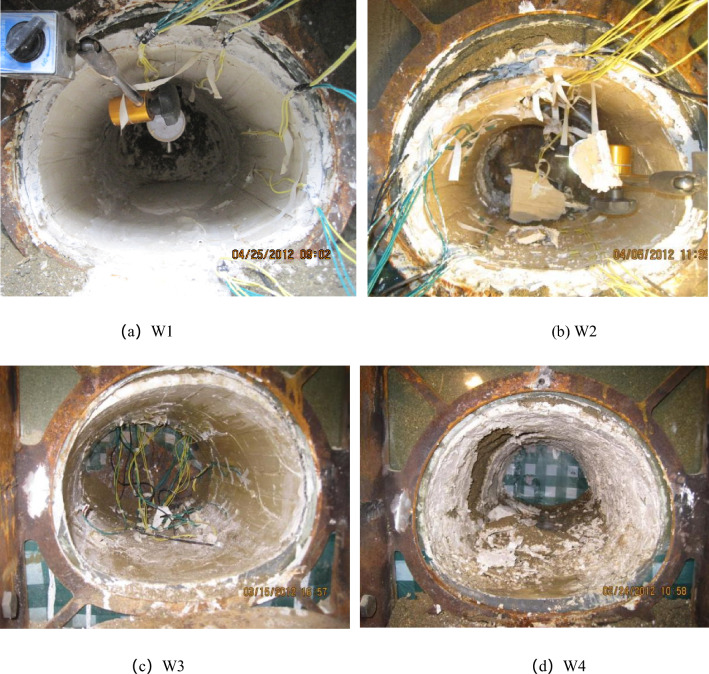
The final state of the conditions W1–W4.

From Fig. 4, it can be seen clearly that the lining under the condition of W2 and W4 is not able to continue to withstand pressure as it has been completely damaged. Meanwhile, under the conditions of W1 and W3, although the deformation is larger, they are finally stabilized.

The appearance and development of secondary lining cracks were in the order of the first arch and the last sidewall, and the shape of the crack is generally longitudinal crack showed in the first, then cracked along the ring. The crack showed in W1–W3 were about the same. Into the late development of the cracks, the W1 and W3 crack width no longer increased, but they showed some new cracks, and the W2 crack width steady increased, in the end, the arch came out, and even the whole secondary lining was collapsing.

### Data analysis of surrounding rock deterioration process

1. Change law of displacement

**Figure 5:**
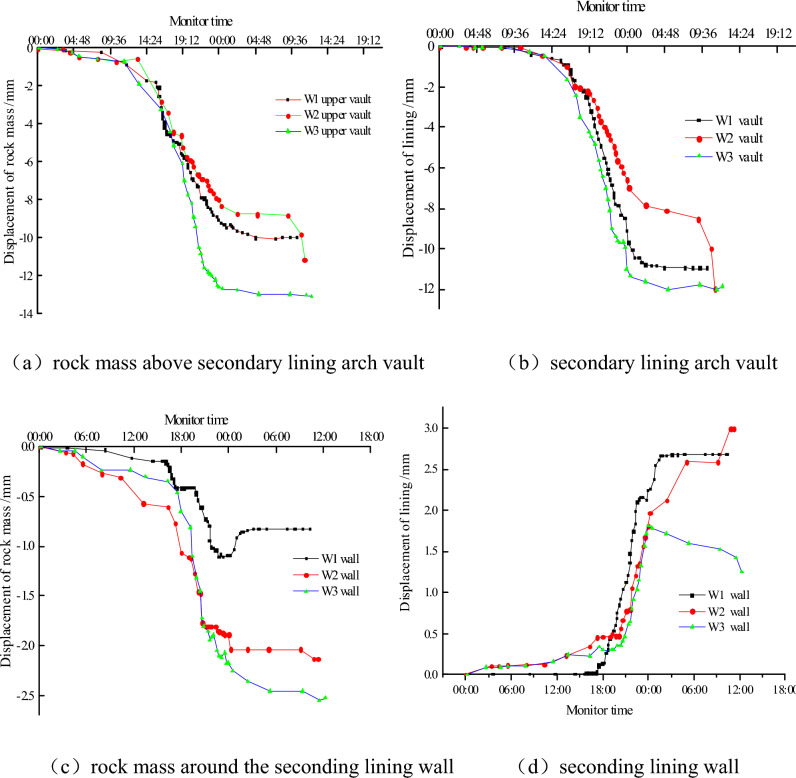
Displacement of rock mass and lining.

Figure 5 is the displacement of the second lining and ground.

In the early stage, the ground displacement is larger than the secondary lining deformation. This is probably related to that there were certain gaps between rock and second lining. When the secondary lining began to crack, the displacement rate of the ground was smaller than the secondary lining deformation rate. The crack of the secondary lining damaged the integrity of the whole structure, and the increase of the deformation rate also shows that the overall performance of the structure was reduced. With the increase of time, the arch vault deformation begin to stabilize after reaching 10–14 cm, but under the condition of W2, the deformation was still continuous and it not be able to carry load anymore.

All of the displacements are in the direction of the inside of the cave. From the view of numerical, the W3 numerical is the biggest, the vertical displacement of the W1 is minimal, and the horizontal displacement of the W2 is minimal. Although the surrounding rock around sidewall displacement was in the direction of the inside of the hole, it moved in the direction of the outside of the cave to meet the deformation coordination of the whole structure. This could be because of the large overall rigidity of the secondary lining.

From the W3 side wall deformation law, we can see that, as the thickness decreases, as the thickness decreases, the increased force of the side wall makes it moved in the direction to the inside of the cave. This could imply that the displacement of the surrounding rock at the sidewall in the W3 is bigger than the displacement of the W1 and W2. The ground displacement of the sidewall of W1 is the smallest as the deformation of the sidewall of the secondary lining to the outside is against the deformation of the surrounding rock.

2. Change rule of surrounding rock pressure

**Figure 6:**
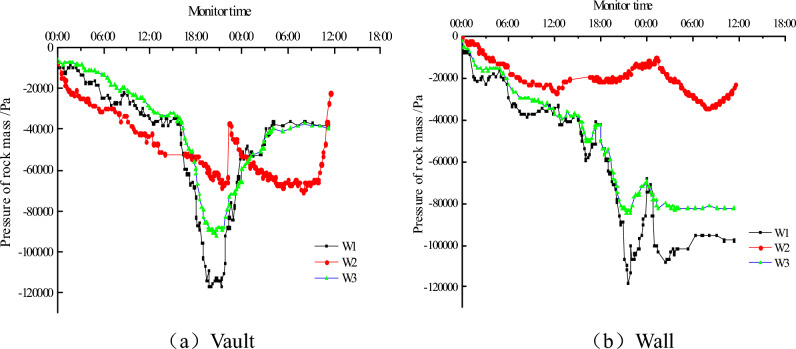
Pressure of rock mass on the lining.

Figure 6 shows the W1–W3 vault and side wall of the surrounding rock pressure curve.

The surrounding rock pressure of secondary lining shows a steady increase in the beginning, with the crack of the secondary lining, the surrounding rock pressure of the arch vault suddenly decreased.

The deformation law of the surrounding rock pressure under W1 and W3 is unanimous, but the numerical value under W3 is smaller. Surrounding rock pressure under the condition of W2 is higher than that of W1 and W3, and there was no significant growth in the latter part. There tends to be a significant reduction in the bearing capacity of the lining with the emergence and development of cracks.

3. The variation law of internal force of lining

**Figure 7:**
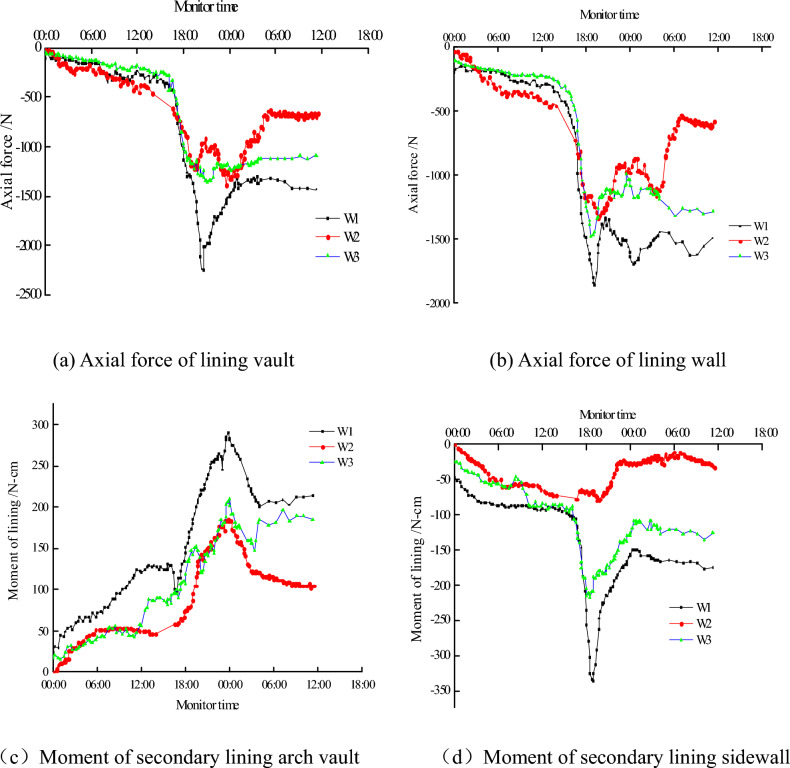
Internal force of lining.

The internal force of lining is the most important design and safety evaluation index. Figure 7 shows the arch vault and the sidewall of the W1 and W2 of the axial force and bending moment curve. The bending moment which drawn to the inner side of the secondary lining is positive, and the axial force is positive if it is under pull.

The whole variation of internal force is similar to that of surrounding rock’s pressure. At the beginning, the internal force increases slowly as the pressure of the surrounding rock increases, and when the pressure of the surrounding rock is so high that it cracks the lining, the value of the internal force of the lining suddenly decreases. With the passage of time, the internal force gradually increases and tends to be stable. From the absolute value, the internal force under W1 appear to be the largest, the values under W1 and W2 are not significant at the initial stage of wall rock degradation, but they are slightly larger than that under W3. With the deterioration of the surrounding rock, value under the condition of W3 increased significantly related to W2.

Although the bending moment increase rate was obviously faster than the increase rate of axial force at the beginning of the deterioration of the surrounding rock, the value of the axial force of the secondary lining is larger than the bending moment’s value. The maximum eccentricity under W1 was 5 mm, the maximum eccentric distance under W2 was 2.9 mm, and that under W3 was 3.8 mm. Then, the secondary lining eccentric compression member, and one side of the secondary lining was in the tension state, which is easily cracking. With the further deterioration of surrounding rock, crack gradually emergence, the increased rate of the bending moment is slowing down, the eccentric distance gradually decreases and stabilizes at about 1.5 mm. That is computed by 0.1 times the thickness of the lining, it belongs to the small eccentric compression member.

4. The variation of internal force of anchor bolt

**Figure 8:**
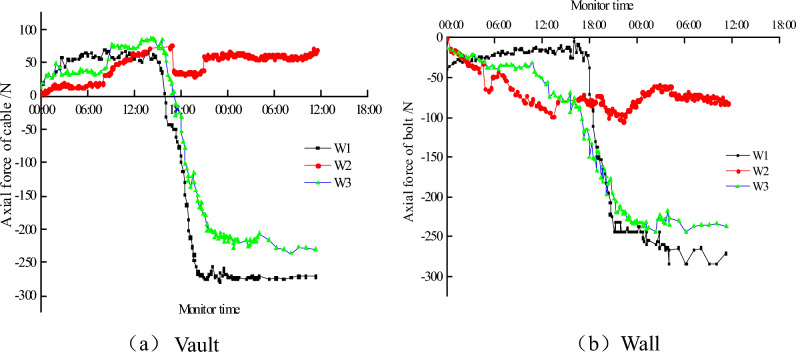
Internal force of bolt.

The Axial force of the anchor bolt was used to judge its internal force. As it shown in Fig. 8.

In the early stages of deterioration of the surrounding rock, the anchor of the arch is tensile, and the anchor of the sidewall is compressed. With the further deterioration of the surrounding rock, the internal force of the bolt increases gradually, and the bolt under conditions of W1 and W3 arch will be changed from tension to compression during the deterioration of surrounding rock. The value under W1 is obviously greater than that under W3.

The bolt of the sidewall is always under pressure. The numerical value of the anchor bolt along with the deterioration of the surrounding rock does not have a significant increase under the condition of W2. Under the condition of W1, the axial force of anchor bolts does not increase significantly at the beginning, but that has experienced a dramatic increase with the deterioration of surrounding rock.

### Test result analysis

In the easily deteriorated surrounding rock tunnel, it is difficult for Plain concrete lining and the non-lining structure to stay stable, for instance, under the conditions of W2 and W4. Driving by the good toughness, the reinforced lining appears to have a larger lining deformation, however, the whole structure did not collapse or fail.

In the early stages of the deterioration of the surrounding rock, the increase in various data is not obvious. This is mainly because water was injected from the top, and the water penetration needs a certain time. With the increase of water injection, the extent and degree of surrounding rock deterioration gradually increased. Meanwhile, the lining deformation, the surrounding rock pressure, lining internal force increased rapidly. When the deterioration of surrounding rock is completed, the lining and deterioration will form a new balance, and the data will be gradually stabilized. If the pressure of the surrounding rock deterioration is greater than the carrying capacity of the lining, the surrounding rock and the lining will not be able to form a new balance. Then, the lining will be damaged, just like that under the conditions of W2 and W4.

In the early stage of the deterioration of surrounding rock, the surrounding rock and the initial support are not close to each other. The initial support and lining not close to each other would lead to the pressure of the surrounding rock subjected to the lining is not uniform. Then, the lining of the eccentricity generally turns out to be large, and the lining is prone to crack if its material tensile capacity appears to be insufficient. With the further deterioration of the surrounding rock, the non-sticking parts will be compacted. That will drive the pressure of the surrounding rock more uniform, and the eccentricity will begin to decrease gradually. Therefore, cracks in the lining could not imply the whole structure is damaged. In other words, it is unreasonable to use cracks as the ultimate state of the tunnel structure.

## Discussion

In the modern world, a large number of weak rock tunnel research mainly concentrated on how to reduce the surrounding rock deformation and the methods to ensure the security and stability in the construction process of tunnel structure. However, there are few studies related to the problems in the process of tunnel construction. Many scholars believe that tunnel structure in the construction period of safety and stability appear to be the most critical one. It is suggested that, when the construction period is safe and stable, the probability of late problems tends to be very low. In fact, many existing tunnel diseases indicate that probability of tunnel structure damage in the operation period should not be neglected. With the appearance and popularization of tunnel structure life-cycle design philosophy, operation safety of tunnel structure became the hot topic of current research.

According to a large number of surveys conducted by the author, the existing tunnel diseases are mainly distributed in the weak surrounding rock. In the construction, the surrounding rock deformation is often accompanied by large deformation. In consideration of such characteristics, in order to limit the deformation of surrounding rock, the second lining is often applied. And it is suggested that the thickness and strength of the second lining are relatively high and that has achieved some good results in previous tunnels’ design and construction.

This paper focuses on the impact of the deterioration of surrounding after the completion of the tunnel construction. Energy dissipation structure theory could be used to explain the problem of surrounding rock deterioration. Energy dissipation structure theory refers to the evolution from disorder to order in the far-from-equilibrium open system. The basic process of dissipative structure formation relates to a far from the equilibrium open system which states through constant exchange of matter and energy with the outside world. When the change of some parameters in the system reaches a certain value, the system could be transit to a non-equilibrium phase with fluctuation.

The process of tunnel excavation and stress evolution at the later stage is the process of energy and material exchange between the tunnel system (including the surrounding rock and supporting structure) and the external environment. Therefore, as a whole, the tunnel system is a nonlinear open system, and the energy dissipation runs through the whole process of the deformation and evolution of the whole tunnel.

The deterioration process of the surrounding rock is a process from elasticity to plasticity. Before the tunnel excavation, the formation is in a stable state, and along with the excavation, the equilibrium state of rock is broken. The elastic deformation is the beginning of the process, there is no macro reversible process and the space has a uniform structure (that is, the disordered structure). Then, the surrounding rock system is still in a state of equilibrium. As excavation continues, the increase in the internal stress of rock mass could cause a large number of microcracks to generate irreversible deformation. With the application of the supporting structure, the whole system is in the process of energy transfer and exchange. Further, the surrounding rock continuously absorbs the energy of the supporting structure and releases energy through the microfracture.

The deterioration process of surrounding rock refers to the process of the deformation and transfer of the surrounding rock's own energy. The supporting structure could be regarded as the energy dissipation structure, which is in the elastic state before the crack. The space is also in the state of disordered structure. When the absorbed energy is beyond the limit of its own, it will release energy to the outside by the way of cracking and the deformation of the supporting structure can also stimulate the release of the deformation energy of surrounding rock. This also could be the reason that the pressure of the support structure increases. With continued deterioration of surrounding rock, the energy will continue to transfer to the supporting structure. The supporting structure can also release energy. That is more than the limit of itself through its own deformation until the final stability is formed.

From the above analysis, the ratio of the numerical value fluctuation and the corresponding structure to the energy appear to be an important index to determine the stability of the structure. This could be computed as (1).

1 $$\gamma_{{{\text{Si}}}} = \frac{{{\text{dD}}_{{\text{i}}} }}{{{\text{S}}_{{\text{i}}} }}$$

*γ*_Si_—Ratio of energy to total energy in the i-th release; dD_i_—The energy released by i-th; S_i_—Total energy in the structure.

The larger the value of *γ*_Si_ tends to be more stable than the tunnel structure. For the early stage of the deterioration of the surrounding rock, the energy before the second lining cracking is the biggest, but the energy released after the first cracking is also very significant. Tunnel structure tends to be unstable at this time. With the development of the crack, the energy continuing release, and the energy release of the new crack is gradually reduced. If the structure of the energy reduction is not obvious, the numerical value of *γ*_Si_ will be convergent and the overall structure appears to be stable. If the energy capacity of the structure is less than the amount of energy released, then, the value of *γ*_Si_ will be enlarged gradually and the overall structure will become unstable.

In order to stabilize the tunnel structure, the approach of reducing the energy of tunnel structure is applied. Toughness refers to the ability of a material to absorb energy after being subjected to force, and materials with high toughness usually have high elasticity and can be deformed without rupturing or breaking under the action of external forces. In practice, the ability to bear the decreasing rate of tunnel structure energy is mainly associated with the toughness of tunnel structure. Meanwhile, the single energy release is mainly related to the stiffness of the tunnel structure. Then, the relationship between resilience and the ability to reduce the rate of commitment tends to be positive. The greater the stiffness of the structure, the more the amount of energy it takes in its initial state, and the energy released by the crack is relatively large. When the toughness is poor, the value of *γ*_Si_ tend to increase easily.

According to the model test applied in this paper, If the development of the surrounding rock is not limited, the initial support will be very easy to destroy as a result of lacking energy capacity. Due to the thicker secondary lining greater rigidity, it could bear more loads. The internal force of the value was significantly higher than the smaller thickness of the secondary lining, and the energy is also larger than the smaller thickness of the secondary lining. In other words, the structure remains stable as a whole as long as the toughness is enough.

To compare the case under the condition of W1 and W2, it could reflect the role of reinforcement in enhancing structural toughness. The concrete lining and the reinforced concrete lining tend to have significant differences in the problem of surrounding rock deterioration. The concrete structure is damaged by the development of cracks and cracks, and the carrying capacity is reduced soon. This could lead to large deformation and cracks, then, which will not be suitable for continue use even though the durability is insufficient. On the other hand, the reinforced concrete lining is consistent with the concrete in the cracking time. Although there is a large deformation, the reinforced concrete can effectively prevent the development of cracks. This could because the integrity of the lining has not been destroyed. This means long-term security is guaranteed.

## Conclusion

By applying the model test, this paper reveals the characteristics of the mechanical evolution of the second lining in the deterioration process of the surrounding rock. The following conclusions are conducted.

1. In the process of deterioration of surrounding rock, a large amount of energy is released. And that is sufficient to cause the destruction of the second lining, this will further affect the overall stability of the tunnel structure, so the toughness of the secondary lining is often the most important indicator of tunnel safety.
2. From the comparison of the deterioration process of the four different types of linings, the addition of steel reinforcement to the concrete lining effectively improved the structural toughness and effectively prevented crack development.
3. Interpreted in terms of energy dissipation theory, a lining with better toughness should be used in order to stabilize the tunnel structure. The lining with better toughness has larger energy, higher bearing capacity, and is better able to maintain the overall stability of the structure.

So it is very necessary to use reinforced concrete lining or reinforced concrete lining with good ductility in the surrounding rock.
